# A Mobile Medical Knowledge Dissemination Platform (HeadToToe): Mixed Methods Study

**DOI:** 10.2196/17729

**Published:** 2020-05-27

**Authors:** Ido Zamberg, Olivier Windisch, Thomas Agoritsas, Mathieu Nendaz, Georges Savoldelli, Eduardo Schiffer

**Affiliations:** 1 Division of General Internal Medicine Department of Medicine University Hospitals of Geneva Geneva Switzerland; 2 Faculty of Medicine University of Geneva Geneva Switzerland; 3 Division of Urology Department of Surgery University Hospitals of Geneva Geneva Switzerland; 4 Department of Health Research Methods, Evidence, and Impact McMaster University Hamilton, ON Canada; 5 Division of Anesthesiology Department of Anesthesiology, Clinical Pharmacology, Intensive Care, and Emergency Medicine Geneva University Hospitals Geneva Switzerland

**Keywords:** clinical skills, clinical competence, clinical practice guidelines, medical education, smartphone, innovation, medical guidance, mobile phone

## Abstract

**Background:**

Finding readily accessible, high-quality medical references can be a challenging task. HeadToToe is a mobile platform designed to allow easy and quick access to sound, up-to-date, and validated medical knowledge and guidance. It provides easy access to essential clinical medical content in the form of documents, videos, clinical scores, and other formats for the day-to-day access and use by medical students and physicians during their pre- and postgraduate education.

**Objective:**

The aim of this paper is to describe the architecture, user interface, and potential strengths and limitations of an innovative knowledge dissemination platform developed at the University of Geneva, Switzerland. We also report preliminary results from a user-experience survey and usage statistics over a selected period.

**Methods:**

The dissemination platform consists of a smartphone app. Through an administration interface, content is managed by senior university and hospital staff. The app includes the following sections: (1) main section of medical guidance, organized by clinical field; (2) checklists for history-taking and clinical examination, organized by body systems; (3) laboratory section with frequently used lab values; and (4) favorites section. Each content item is programmed to be available for a given duration as defined by the content’s author. Automatic notifications signal the author when the content is about to expire, hence, promoting its timely updating and reducing the risk of using obsolete content. In the background, a third-party statistical collecting tool records anonymous utilization statistics.

**Results:**

We launched the final version of the platform in March 2019, both at the Faculty of Medicine at the University of Geneva and at the University Hospital of Geneva in Switzerland. A total of 622 students at the university and 613 health professionals at the hospital downloaded the app. Two-thirds of users at both institutions had an iOS device. During the practical examination period (ie, May 2019) there was a significant increase in the number of active users (*P*=.003), user activity (*P*<.001), and daily usage time (*P*<.001) among medical students. In addition, there were 1086 clinical skills video views during this period compared to a total of 484 in the preceding months (ie, a 108% increase). On a 10-point Likert scale, students and physicians rated the app with mean scores of 8.2 (SD 1.9) for user experience, 8.1 (SD 2.0) for usefulness, and 8.5 (SD 1.8) for relevance of content. In parallel, postgraduate trainees viewed more than 6000 documents during the first 3 months after the implementation in the Division of Neurology at our institution.

**Conclusions:**

HeadToToe is an educator-driven, mobile dissemination platform, which provides rapid and user-friendly access to up-to-date medical content and guidance. The platform was given high ratings for user experience, usefulness, and content quality and was used more often during the exam period. This suggests that the platform could be used as tool for exam preparation.

## Introduction

The teaching of clinical skills and choice of management plans are important cornerstones in medical schools’ curricula [1]. Skills like medical and personal history taking, physical examination, and procedural skills are taught in parallel to more theoretical aspects and prepare the medical student for his or her clinical years. A medical student is expected to be able to examine patients displaying a large variety of complaints, as well as to perform many different medical procedures. Throughout medical school, students’ clinical skills are evaluated and in the Switzerland medical federal licensing examination, passing an Objective Structured Clinical Examination [2] test is mandatory in order to become a fully certified physician. Like any skill, clinical skills improve with experience, repetition, and constructive feedback provided by supervisors. Nevertheless, students rely on references (ie, documents and videos) to prepare themselves for the clinical environment [3,4]. Even though a multitude of references do exist for clinical skills, the information is vast and scattered between a large number of sources, and finding high-quality and validated references can be a time-consuming and frustrating process [5,6].

In the clinical environment, students and residents rely on clinical practice guidelines and recommendations to choose a treatment plan for a patient, which are, as mentioned, not always readily available [6]. Finding optimal guidance regarding clinical skills, procedural skills, or patient management is challenging for all clinicians. It is even more so for students and postgraduate trainees, who seek not only sound and trustworthy guidance [7,8] but also perhaps the most didactic and one that fits the expectations of their educators and senior clinical staff. Thus, learners can benefit from a dissemination platform whose content is authored or endorsed by their local faculty, educators, and senior hospital staff.

Moreover, the use of smartphones and other mobile devices in the medical environment, commonly termed mobile health (mHealth) [9], has increased rapidly throughout the last several years [10-12]. mHealth can offer not only patient-centered solutions for chronic illness management, behavioral change, or self-monitoring [13-22] but also learning opportunities for pre- and postgraduate medical education [23]. The online environment plays an important role as well in continuous education, and health professionals rely on easy-to-access and high-quality medical content to improve patient care [24-27]. Furthermore, it was shown that health professionals favor significantly well-known targeted medical resources to more general web browsing in their search for what they perceive as validated medical content [28].

This increasing use of online resources and smartphones and the need for easy-to-access, validated, and high-quality medical information urged the development of HeadToToe, a mobile platform intended for the dissemination of medical knowledge and tailored guidance for pre- and postgraduate health professionals.

The purpose of this article is to describe the architecture of the tool and its potential strengths and limitations compared to existing tools, as well as to present preliminary evaluation of usage statistics and user experience. Our hypothesis at this stage of development is that users would perceive our platform as a rapid and user-friendly way to access up-to-date medical content and guidance, and that its content, being validated by local senior educators, would be therefore perceived as trustworthy and useful, both for education and practice.

## Methods

### Platform Description

**Figure 1:**
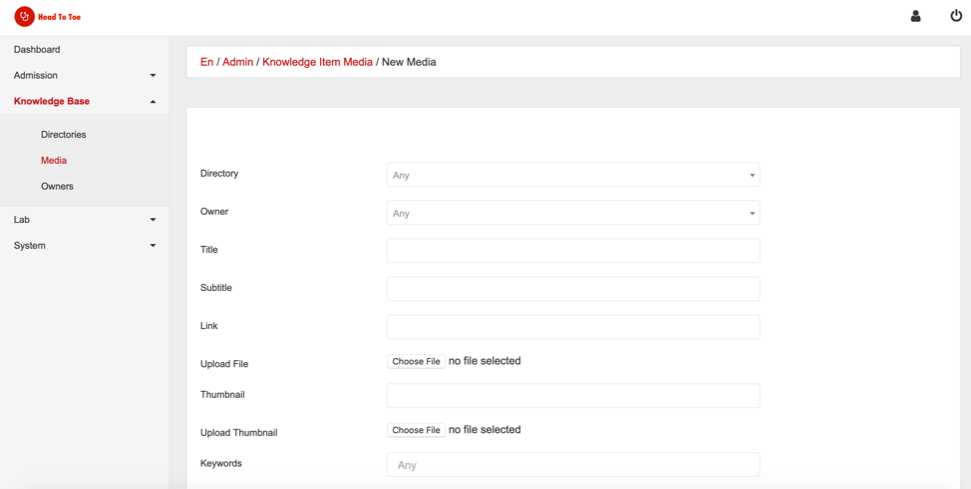
The administration interface of the HeadToToe platform. Educational content managers can use the interface to insert, update, and delete new content. Through the interface, administrators can also create the admission checklist for the admission checklist section as well as the laboratory values section.

The HeadToToe [29] platform consists of an iOS and Android front end that is compatible with iPhones, iPads, Android phones, and tablets. Both front ends connect to the same back-end server, which includes server-side code and a database allowing the centralization of all content and easy updating of the platform. The platform is managed through an administration interface (see Figure 1), which is hosted on the same server as the database. A blank version (ie, content-free version) of the platform can be made available to other institutions for the purpose of academic collaborations. The current operational platform is in French, but the platform does support a multilingual environment.

### Back End: Database and Administration Interface

#### Basic Architecture

The server is hosted on Heroku and contains the database, the code, and the application programming interface (API) for its administration. The API and the administration interface were written with Ruby on Rails, version 2.5.3 (MIT). The administration interface provides a user-friendly interface for adding, deleting, and editing content. Access to the administration interface is made through a weblink, restricted by a username and a password for educational content managers (ECMs) (ie, faculty educators and senior hospital staff managing content in HeadToToe).

#### Content Management

The cornerstone of the platform is that the ECMs are responsible for identification, endorsement, and updating of the content for the medical field under their responsibility. This provides the validation that learners need to be able to trust that the content provided by the platform meets their educational needs. The users themselves do not have access to the management interface and cannot upload their own content. Each time the app is launched or refreshed by the users, new or updated content is highlighted with a specific icon.

#### Content Metadata

Each content item has several attributes, including title, subtitle, search keywords, link, thumbnail, update date, and expiration date. An item can be a link to a file, a video, or a website. In addition, each item’s metadata includes the full name and contact details of the ECM, providing the users the possibility to communicate with content managers.

#### Content Item Responsibility

The content item responsibility can be defined by clinical field, subfield, or by individual content items. For example, the head of neurology could be the ECM for the whole neurology section or could delegate the responsibility for certain subfields or items to other senior medical staff or educators.

#### Duration of Validated Content and Updating Mechanism

Medical knowledge and guidance, whether international, regional, or local, is always evolving. For each item, the ECM selects an expiration date, thus defining the duration over which the content will be accessible to users. Automatic notifications signal the author when the content is about to expire, thus promoting its timely updating and reducing the risk of using obsolete content. Namely, 2 weeks and 24 hours before a content item reaches its expiration date an automatic email will be sent to the ECM to remind him or her about the expiration of the specific content item. The ECM will then be able to decide if the item is still valid, thus prolonging its availability on the platform. If the item is considered not valid, the ECM may update it with a newer version or delete it. Eventually, expired items will be deleted automatically after a predefined period if no action confirms the validity of the resource.

### Front End: User Interface

#### The Front End of the iOS and Android App Includes Several Sections

##### The Knowledge Base

**Figure 2:**
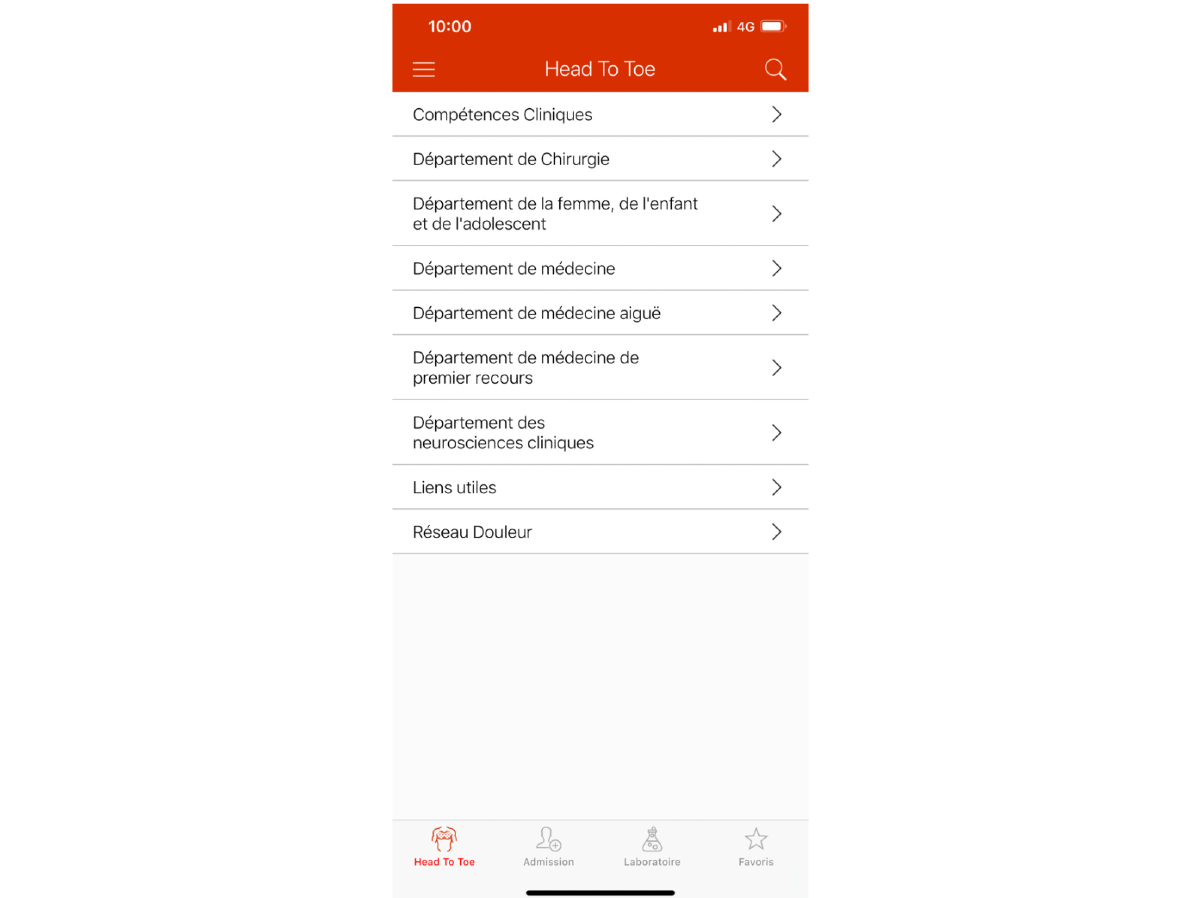
The knowledge base of the HeadToToe platform (in French). Users can navigate through different medical specialties organized as folders.

The knowledge base is the heart of the app (see Figure 2) and contains the medical content and guidance organized by clinical specialty with folder hierarchy defined through the administration interface by the ECM of each section. Folder hierarchy is flexible and can differ between clinical fields. This section allows the user to navigate through each medical field’s sections and find the needed content. After navigating to the desired section, the user can access files or media (ie, videos or website links). An information button is available for each document and media item. A click on the button will open a pop-up view containing the item’s metadata: contact details for the ECM, last update date, and expiration date. The expiration date will show in green within the duration of its availability and in red when it is expired; it will stay red until it is either updated, manually deleted by the ECM, or automatically deleted after a predefined period. For documents, a download button is present and allows download and offline use.

##### Admission

**Figure 3:**
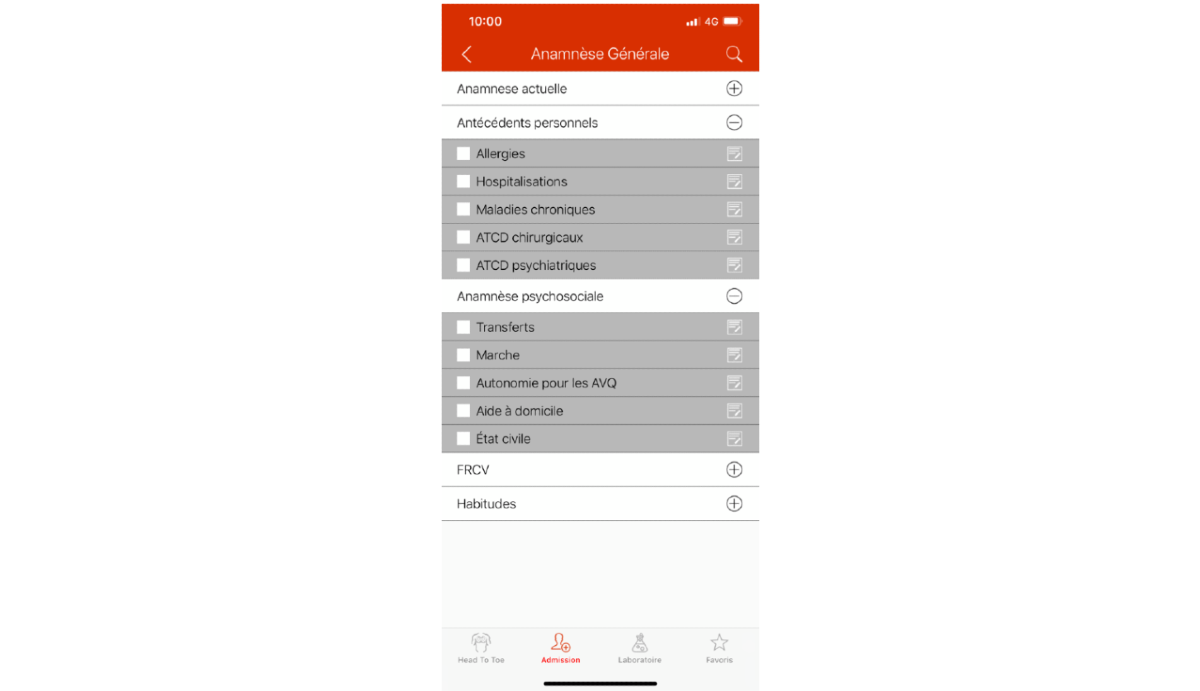
History-taking and clinical examination checklist section in the HeadToToe plaform (in French). Users can consult the list, add comments, and create a PDF with all checked items and added information.

In this section (see Figure 3), users can find an extensive checklist with questions to be asked during history-taking of a patient and actions to perform during clinical examination. Items can be checked, comments can be added to each item, and the user can generate a PDF containing checked items and comments.

##### Laboratory Values

**Figure 4:**
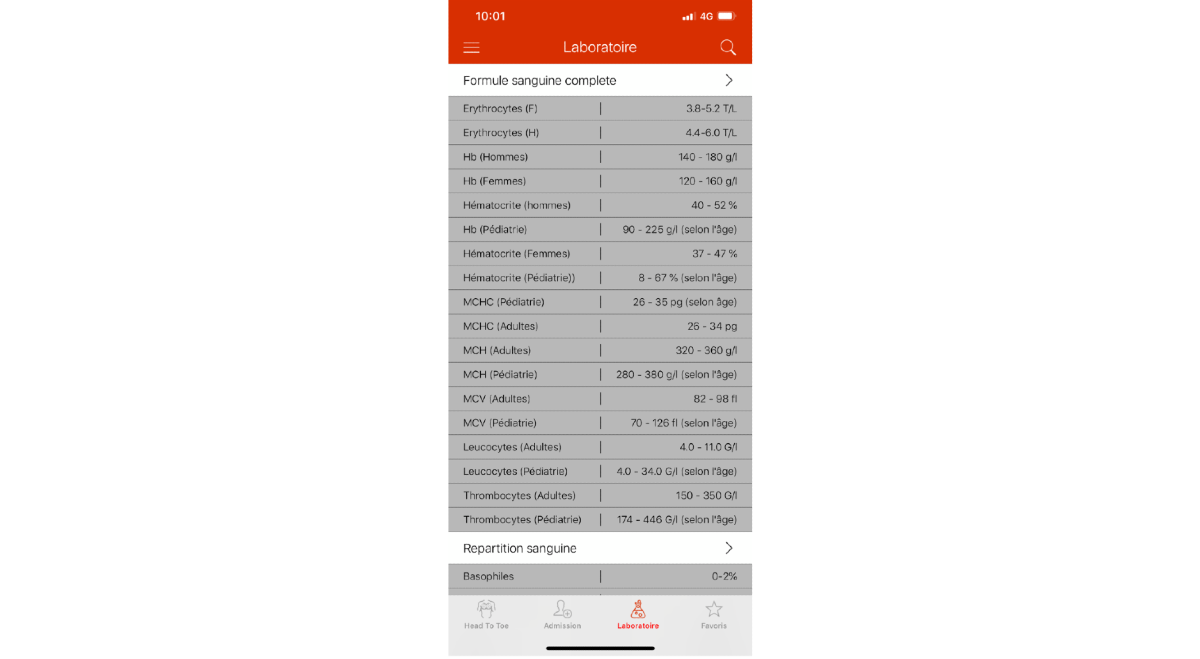
Laboratory values in the HeadToToe platform (in French). Users can consult basic laboratory values validated for local reference values.

This section (see Figure 4) contains local reference values for frequently used laboratory values, such as complete blood count, chemistry, basic metabolic panel, arterial blood gas, and more.

##### Favorites

**Figure 5:**
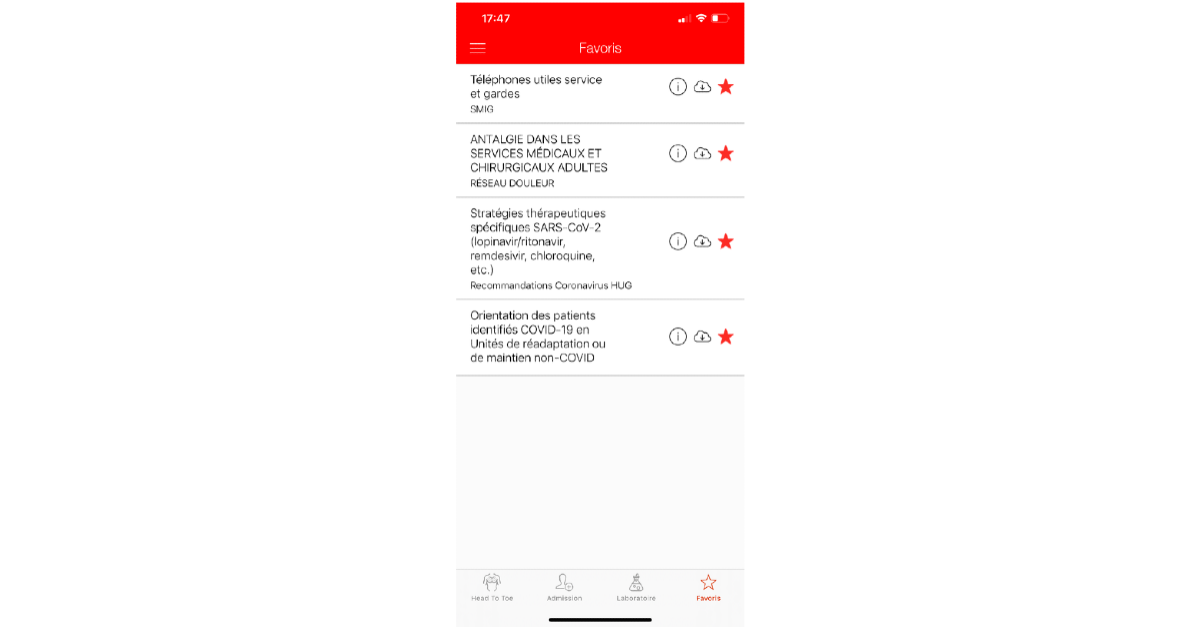
Favorites screen in the HeadToToe platform (in French). Through this screen, users can quickly access marked content.

**Figure 6:**
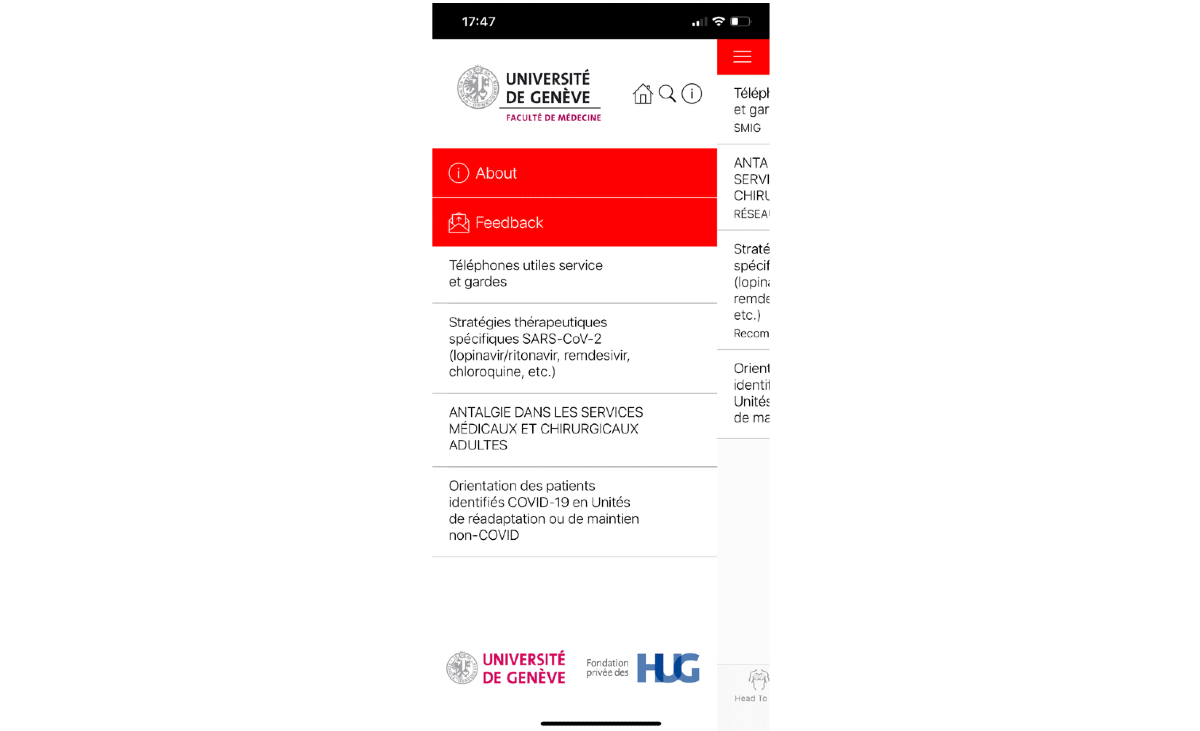
Side menu in the HeadToToe platform (in French). Through this menu, users can quickly access their favorite content and the *About* menu. Users may also access the Feedback screen and send feedback and questions concerning app use and content.

Documents in the knowledge base can be marked as favorites. This section shows the list of favorites and allows users to unmark them (see Figure 5). Favorite documents can also be found on the side menu of the app (see Figure 6).

##### Search Screen

**Figure 7:**
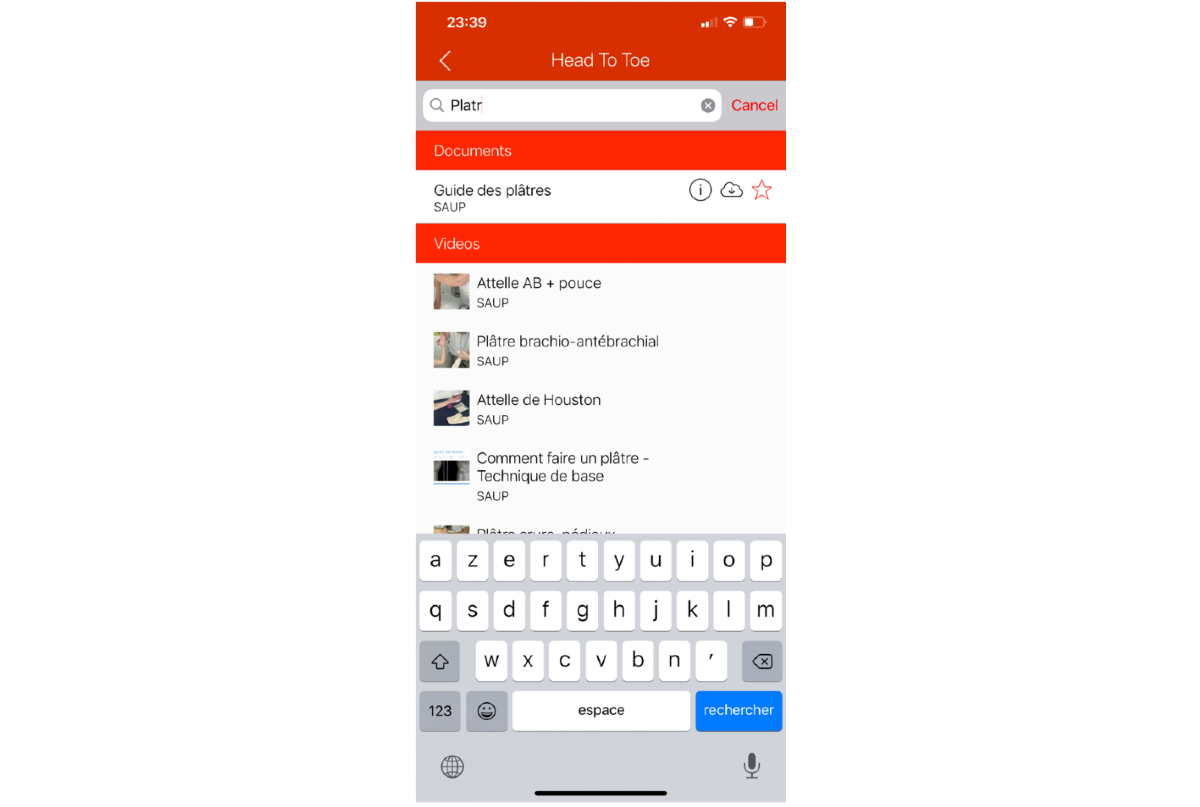
The search screen of the HeadToToe platform (in French). Users can search through all types of content from this screen and quickly access needed information.

The search screen (see Figure 7) is accessible throughout the app and allows a transversal search through all content (ie, documents, media, and lab values) for quick access to needed content. A search is made by the item’s title, subtitle, and keywords defined in the administration interface to improve search accuracy.

### Implementation and Dissemination of the App in the Institution

Target users of the platform are medical students in the Faculty of Medicine at the University of Geneva and postgraduate residents from the University Hospital of Geneva, Switzerland. The app is currently available for download exclusively through a private website in order to preserve the content’s copy and the authors’ rights of local protocols as well as content imported from external sources. The iOS app is distributed through Apple’s Enterprise distribution methods. The Android app is distributed through the same private website with an APK (Android Package Kit) file. Students and physicians have separate versions for distinct statistical analysis. A student does not have the credentials to download the physicians’ version, and vice versa, to ensure statistical separation.

In the pregraduate level, the app was implemented at the University of Geneva in 2015 as a beta version and the final version was released in March 2019. The medical curriculum consists of a fully integrated bachelor’s, master’s, and medical degree 6-year program. The teaching of clinical skills starts from as early as the second year onward; clinical skills are taught in parallel and with relation to basic sciences studies until the end of the sixth year [30]. Therefore, the target population was medical students from the second to the sixth year of medical school, which includes roughly 750 students at a given time. Since the launch of the final version of the app in March 2019, to date, the app was downloaded 622 times, counting multiple user devices.

At the postgraduate level, we first performed a pilot test from November 2018 to February 2019 and disseminated the app primarily in the Division of Neurology at the University Hospital of Geneva, Switzerland. In March 2019, we launched the final version of the app and made it progressively available to the Divisions of General Pediatrics, Pediatric Emergency, Neonatology, Neurosurgery, Pre-Hospital Emergency Care, Anesthesiology, Urology, Nephrology, Hematology, Diabetology, and Primary Care. Our roadmap includes the implementation of HeadToToe in more divisions, and eventually the whole institution, in coordination with all relevant medical leadership. So far, the app was downloaded 613 times by a total of approximately 1900 doctors from the above-mentioned divisions in our institution (see Table 1).

**Table 1:** User distribution between university students and hospital medical personnel since the launch of the final version of the HeadToToe platform in March 2019.

Users and devices		App downloads^a^, n (%)	Potential users^b^, n	Potential yearly renewals^c^, n
**University of Geneva students**				
	Total	622 (100)	750	150
	Users with Apple devices	430 (69.1)	N/A^d^	N/A
	Users with Android devices	192 (30.9)	N/A	N/A
**University Hospital of Geneva health professionals**				
	Total	613 (100)	1900	300
	Users with Apple devices	409 (66.7)	N/A	N/A
	Users with Android devices	204 (33.3)	N/A	N/A

^a^Includes multiple-device downloads by the same user.

^b^Represents an estimated number of members of the target audience in the mentioned institutions.

^c^An estimate of new institutional students or employees, based on mean number of new medical students per year as well as mean number of new medical staff in our institution.

^d^N/A: Not applicable.

### Educational Content Managers: Authoring or Endorsing Content

To ensure validated and high-quality content to the best of our abilities, we contacted senior physicians in different medical fields in our institution. Pregraduate content was chosen by head faculty members responsible for clinical skills teaching, and postgraduate content was selected by either heads of divisions and units or by other physicians from the respective divisions or units to whom the mission was delegated. Each of these specialists was responsible and contributed to his or her field’s section in the platform with international and local content that he or she deemed updated, validated, and useful for his or her fellow colleagues. Each department, division, and unit in the institution can have its own section in the app without any content-type or size restriction. The goal of combining pre- and postgraduate content was to allow continuous usage of the platform from the student level, through residency, and the rest of medical training.

### Platform Development Process

The platform’s concept emerged from an urgent need and difficulties mentioned above by some of the authors to find high-quality and locally validated information concerning clinical skills during their medical university years. Several members of our team have dual training in computer science and medicine, which puts them in a position to be both code and architecture developers, as well as end users of the platform. This position made the design of the app and its architecture user-centered from its basic core, as we were not only developing a product but a tool that we ourselves use in our daily practice.

To ensure an even more user-centered development process, before launching the final version, we held several trial periods for pre- and postgraduate users in order to try and understand each group’s specific needs and to receive feedback. In addition to the postgraduate trial period described earlier, a beta version was launched in 2015 and used by 93 fifth-year medical students in the University of Geneva before distributing the app to the rest of the faculty. In addition, both users and ECMs have the possibility to send us feedback and ideas directly from the app and from the administration interface. Automatic utilization statistics allow us to identify the most-used type of content and, thus, to add similar content as well as to eliminate unused content. Lastly, as discussed further in the Platform Assessment section, satisfaction surveys were sent to platform users with the possibility to write free-form text feedback.

These measures allowed us to improve user experience, fix bugs, and add requested features and content as suggested by medical students and health professionals during each step of the development.

### Platform Assessment

Evaluation of the platform utilization and usage was made in two different ways. First, a factual analysis was conducted with data obtained from statistical collection by Yahoo’s Flurry Analytics to allow quantitative analysis of the utilization of the tool. Four different Flurry API keys were created to distinguish between iOS and Android apps and between medical students and physicians. Collection was made continuously, anonymously, and automatically.

Events sent to Flurry by the iOS and Android apps were the title of the item, the medical field it belongs to, and whether it was accessed by search or directly through the main knowledge base. Statistics are separate for medical students and doctors. This allows us to record summary statistics about how many times each item was used. Flurry automatically collects information about the usage of the app, including the number of active devices per day, number of sessions per day, number of sessions per device per day, time of usage per device per day, median session length, and more. The user’s journeys are also recorded, which means we are able to follow each user’s session and actions made in order the find needed information as well as the amount of time spent to retrieve it.

A second assessment focused on user satisfaction, and qualitative utility was conducted through a survey. One survey was addressed to all medical students, from the second to the sixth year of medical school, and another one was addressed to hospital physicians and nurses from the Division of Neurology, as they were the first to use the app during a trial run. The surveys focused on user experience, general usefulness of the app, and the relevance of the content (see Multimedia Appendix 1 for survey questions). Surveys sent to both students and physicians were identical.

### Statistical Analysis

*P* values were calculated using unpaired *t* tests for continuous quantitative variables. Calculations were made using Stata statistical software, version 16 (StataCorp). *P*<.05 was considered statistically significant.

## Results

### User Demographics

As automatic statistics collection was anonymous, we do not have exact demographic knowledge regarding age and sex of users. We can estimate that the mean age for university medical students is between 20 and 30 years and that for medical doctors is between 25 and 65 years. All users live in Geneva, Switzerland, or its surroundings, and are either medical students at the University of Geneva or health professionals (ie, doctors or nurses) at the University Hospital of Geneva. Distribution of downloads between the university and the hospital, as well as between iOS and Android devices, is summarized in Table 1.

### Pregraduate Analysis

#### Assessment of Utilization

**Figure 8:**
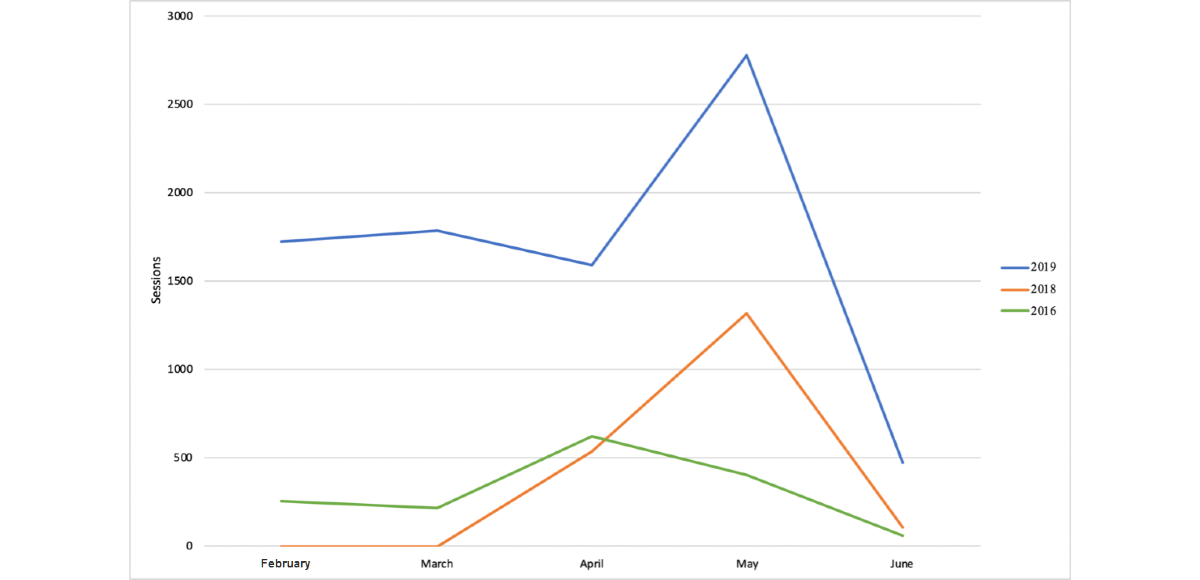
Exam period usage patterns from 2016 to 2019. The exam period is usually at the end of the spring semester, either at the beginning or end of May each year.

In the pregraduate level, during the period from March to June 2019, a total of 251 students downloaded the app (iOS and Android combined). There was a significant rise in daily users and usage time with an average of 24.5 (SE 1.8) students per day during the exam period, compared to 16.5 (SE 1.9) from March to April 2019 (*P*=.003) (see Table 2). This resulted in an average of 8.2 (SE 0.8) minutes per day during the exam period compared to 5.1 (SE 0.5) during the control period (*P*<.001) (see Table 2), and almost double the total number of sessions during the exam period as compared to the months before (see Figure 8). Number of sessions per day increased significantly as well, with a mean of 89.5 (SE 8.3) sessions per day during the exam period, compared to 56.7 (SE 5.4) sessions per day during the control period (*P*<.001) (see Table 2). The median session length, which may reflect the time each user needs to find the requested content, was 35 seconds. This fact can be cross-referenced with *user journeys* gathered by Yahoo’s Flurry Analytics for each user’s session, which shows the exact navigation path each user made through the app and time spent. During this period, 3756 documents and 1570 videos were consulted (see Table 2).

**Table 2:** Pregraduate assessment of utilization during the control period compared to the exam period within 251 HeadToToe app downloads among medical students at the University of Geneva, Switzerland.

Assessment measure^a^		Exam period (May 2019)	Control period (March and April 2019)	Difference	*P* value^b^
**Daily activity, mean (SE)**					
	Active devices	24.5 (1.8)	16.5 (1.9)	8.0 (2.8)	.003
	Minutes per user per day	8.2 (0.8)	5.1 (0.5)	3.1 (0.95)	<.001
	Total sessions per day	89.5 (8.3)	56.7 (5.4)	32.8 (9.5)	<.001
**Content usage, n**					
	Documents consulted	1451	2305	N/A	N/A^c^
	Videos consulted	1086	484	N/A	N/A

^a^Data were from automatic statistics collection with Yahoo’s Flurry Analytics.

^b^Calculated with an unpaired *t* test for continuous quantitative variables.

^c^N/A: Not applicable.

#### Assessment of Utility

In the pregraduate level, 138 students answered the survey. Out of a top score of 10, students rated the app with a mean score of 8.2 (SD 1.9) for user experience, 8.1 (SD 2.0) for usefulness, and 8.5 (SD 1.8) for the relevance of content (see Table 3). A total of 39.1% (54/138) of students considered the video section as the most useful and 42.0% (58/138) considered the document section as the most useful. A total of 48.6% (67/138) of students said they would like to see more procedural skills videos and 30.4% (42/138) said they would like to see more clinical scores.

**Table 3:** Pre- and postgraduate assessment of utility.

Institution and users	N	User experience, mean (SD)^a^	Usefulness, mean (SD)^a^	Relevance of content, mean (SD)^a^
University of Geneva medical students	138	8.2 (1.9)	8.1 (2.0)	8.5 (1.8)
Division of Neurology, University Hospital of Geneva physicians	28	7.8 (2.2)	8.6 (1.7)	8.5 (1.5)

^a^Values represent weighted mean scores on a 10-point Likert scale survey answered by users at both institutions.

### Postgraduate Analysis

#### Assessment of Utilization

At the postgraduate level, the app was downloaded 98 times, counting multiple devices per health professional, during the trial period of 3 months (ie, between November 2018 and February 2019). Regarding usage, there was an average of 10.1 (SD 5.1) users per day for an average of 8.1 (SD 8.6) minutes per user per day with a mean of 55 (SD 35) sessions per day (see Table 3). Median session length was 29 seconds. During this period, 6494 files and 144 videos were consulted (see Table 4). Documents viewed were mainly local guidance for acute stroke management.

**Table 4:** Postgraduate assessment of utilization during a trial period among health professionals in the Division of Neurology at the University Hospital of Geneva, Switzerland.

Assessment measure^a^		Mean (SD)	Range^b^	Total, n	Mean per month (SD)
**Daily activity**					
	Active devices per day	10.1 (5.1)	2-26	N/A^c^	N/A
	Minutes per user per day	8.1 (8.6)	0.8-66	N/A	N/A
	Total sessions per day	55.0 (35.5)	6-162	N/A	N/A
**Content usage**					
	Documents viewed	N/A	N/A	6494	2134 (480.5)
	Videos viewed	N/A	N/A	144	48 (12.2)

^a^Data were from automatic statistics collection with Yahoo’s Flurry Analytics between November 2018 and February 2019.

^b^Represents minimum and maximum values for reported parameters.

^c^N/A: Not applicable.

#### Assessment of Utility

A total of 28 health professionals answered the survey (see Table 3). Out of a top score of 10, user-friendliness was rated with a mean score of 7.8 (SD 2.2), usefulness had a mean score of 8.6 (SD 1.7), and content relevance had a mean score of 8.5 (SD 1.5). When asked for the most useful section of the app, documents were elected at 100%, compared to admission, videos, and laboratory values. When asked for further content addition, 71% (20/28) wanted to have additional protocols, 21% (6/28) asked for clinical scores, and 7% (2/28) elected additional clinical skills videos as the most useful.

## Discussion

### Principal Findings

Finding easy-to-access, high-quality, and validated evidence-based medical content is a challenging task. The use of the online and mobile environment is increasingly growing and becoming a relevant tool for pre- and postgraduate medical education and clinical practice. Health professionals tend to favor the use of targeted medical online and mobile resources as compared to nonspecific web browsing. In order to address these difficulties and to adapt to modern medical content–consuming habits, we have developed the HeadToToe platform.

Medical students and doctors using the app found our platform to be user-friendly and generally useful, and they perceived the content as practical and relevant for daily clinical practice. For example, during a pilot implementation phase in the Division of Neurology, 6500 documents were consulted in 3 months (ie, about 72 per day).

Continuous and automatic user-based statistics, as gathered in our platform, can provide understanding of the students’ learning process and helps identify the most frequently used content items. During an evaluation period of 3 months since the distribution of the final version through March and June 2019, as well as statistics collection from the beta version since 2015, we noted daily constant use of the app with increased activity during exam periods with the same activity patterns for the last 4 years (see Figure 8). Usage in 2017 is not shown as no statistics were gathered due to further development of the platform. We noticed a progressive increase in use during the month preceding the exam period and were able to identify frequently used content during this period. These findings can allow future focus on students’ needs and studying habits, as frequently used content can be further developed. These findings might also suggest that the platform may be used as a tool for exam preparation, specifically in the clinical skills field.

Numerous online and mobile platforms exist and provide highly validated and high-quality medical information. Geeky Medics [31] and Bates’ Guide to Clinical Examination and History Taking [32] are examples of highly used and validated online and mobile platforms that provide access to clinical skills material. The MDCalc website and app is an example of a useful tool for clinical scores calculation [33]. UpToDate and PubMed are other tools used for retrieving evidence-based medical knowledge and treatment plans [34]. These sources are just a few among others, and their use and trustworthiness is widely recognized. Each of these tools provides quality information about a specific aspect of medical knowledge and they are complementary with regard to a specific clinical question [35].

However, local institutions often lack dissemination for clinically relevant content endorsed by local medical educators. Different file managers, such as Dropbox and Google Drive, can be used [36,37] but have obvious limitations. For example, they lack specific medical-relevant structure and user experience and do not provide specific solutions for obsolete content management.

In contrast, our platform has several potential strengths. HeadToToe provides an easy solution for the dissemination of selected medical references within an institution. Content selection is educator driven, which means that ECMs from each medical field in a given institution can be responsible for content management and endorsement, thus ensuring content validation and quality. Information can be retrieved from several validated knowledge bases and is not restricted to a single source. Users can then easily access information from a variety of medical fields through a mobile app, and frequently used content can be tagged and downloaded for offline use. Through the administration interface, adding and updating content is simple, and users will only have access to the most recent version of an item and are automatically notified of the arrival of new content. Moreover, HeadToToe contains a programmatically determined expiration date for each item, which ensures up-to-date content by automatically notifying the ECM when content is about to expire and requires new validation or updating of the item. When no action is taken, expired items will be automatically deleted. This is particularly important in the medical environment where evidence is evolving constantly, and this feature provides a continuously updated platform avoiding the dissemination of obsolete medical content. This feature would be harder to achieve with a simple file manager, as obsolete content is difficult to identify, manage, and suppress and would require a manual updating system. Furthermore, the platform provides metadata for each item, displaying information and contact details of the ECM as well as visibility of the updated date and expiration date.

The centralization of institutional information within a single tool may help with the integration of new medical staff at any stage of their medical career by providing quick and easy access to local practice guidance and practical information, such as important phone numbers, call schedule, and more. For example, we observed that including both pre- and postgraduate material seems to facilitate implementation of the platform, as medical students are exposed to the platform early in their curriculum and continue to use it during clinical rounds and after graduation. This can help them transfer and apply knowledge and skills in the clinical environment and promote continuous education.

From an educational and academic point of view, the tool provides automatic monitoring of content usage and user activity and can provide information to ECMs about content relevance and learners’ needs. Usage data gathered may provide interesting insights for research in the field of medical education. Another advantage of the platform’s unified architecture is that it also offers a simple way to disseminate local medical knowledge to other academic or private partners. Indeed, we are developing targeted versions of the app, which can be customized for specific medical content intended for external use.

Data collected using the platform could help in presenting more evidence for the utility of mHealth solutions in clinical practice. In fact, while many mobile solutions targeted to health professionals are being used on a daily basis, evidence is still lacking concerning their impact on clinicians’ adherence to validated guidance and on the clinical impact on reducing unwarranted variation in practice. By monitoring user activity and content usage patterns, and linking it with clinical indicators, we might be able to present more concrete, real-world data supporting the use of mobile platforms in clinical settings.

The platform might present a cost-effective and ecological solution [38,39] for knowledge dissemination, as it may make obsolete the need for printing medical and practical information for new and current staff members, even more so for information that is updated frequently. In addition, the platforms’ use might present as time-efficient and, therefore, cost-saving for health professionals, as knowledge is centralized and rapidly accessible within a mobile app, thus eliminating the need to find an available computer and to browse the web. The economic and ecological impact of this type of intervention was not yet studied and would be of better value when the platform is fully deployed within the institution.

The platform presents several limitations. The first and main limitation is that it requires the validation and triage of a large amount of medical content as well as the coordination between several educators. The platform also requires content-quality check, which could become time-consuming, as more content accumulates on the platform. The designation of an ECM is a crucial part of the process and does require active participation in the creation of the platform. Nevertheless, institutions are often required to locally adapt or endorse the use of international guidance and to translate it into local practice due to differences in populations and local resources. Thus, triage and centralization of medical content can be useful for identifying existing content and promoting it, as well as identifying fields where content and evidence is lacking and need to be addressed in a given institution. Our experience and the uptake in our institution has so far been very positive but remains to be further assessed. Implementation and evaluation would also need to be assessed in other clinical environments and institutions.

To date, we did not include patients in our platform development and design process, as they were not the target users of the mobile app. Nevertheless, as there is growing evidence for the importance of patients’ input in mHealth interventions, especially for patient-centered resources [19,40-42], inclusion of patients in future developments could be of interest, specifically concerning their perception of the utilization of mobile devices by health professionals in daily practice.

The quality of the content can also be criticized as subjective and be a matter of debate. Indeed, we do not offer a technological solution for the measurement of the content’s quality. However, ECMs, as mentioned, are senior medical staff and specialists who are, by definition, responsible for local strategies, medical education, and treatment plans. Thus, content quality and relevance are guaranteed by the ECMs’ institutional roles. Moreover, the app has the merit of making validated content transparent to all partners and, thus, helps identify either information needs or conflicting guidance on similar topics. Institutions and local ECMs would still need to use sound methods for critical appraisal of content to include. For content that amounts to recommendations for clinical practice, ECMs should use trustworthiness criteria, such as the ones published by the Institute of Medicine [8,43] or by leading experts, such as the GRADE (Grading of Recommendations Assessment, Development, and Evaluation) Working Group [44]. Finally, all online content needs to meet copyright regulations, both for published content as well as for locally created content.

### Conclusions

The HeadToToe platform allows medical educators to create a validated, high-quality, and up-to-date reference platform for simplified pre- and postgraduate medical education knowledge dissemination, to the benefit of their students and medical staff. It is built for easy and quick access. Users found the app to be user-friendly, relevant, and useful for clinical practice. Implementation in different universities and clinical settings would be the next natural step for assessing its relevance in broader settings and scalability. Utilization patterns should be further examined in light of students’ and residents’ information needs and learning habits, both as a tool for exam preparation and for daily clinical activity. The potential impact on the reduction of unwarranted variations in practice, quality of care, and economic outcomes should be further studied, and randomized trials could compare the use of such integrated dissemination platform to current available tools.

## Appendix

Multimedia Appendix 1 Survey questions.

## Abbreviations

API: application programming interface
APK: Android Package Kit
ECM: educational content manager
GRADE: Grading of Recommendations Assessment, Development, and Evaluation
mHealth: mobile health
