# Supplementary material for: A Mobile Medical Knowledge Dissemination Platform (HeadToToe): Mixed Methods Study
Source: JMIR Med Educ. 2020 May 27;6(1):e17729. doi: 10.2196/17729 (PMC7287749; doi:10.2196/17729)
Supplement: Multimedia Appendix 1 [file mededu_v6i1e17729_app1.docx]

**Survey questions HeadToToe**

1. On a scale of 1 to 10, Is the application easy to install?
2. On a scale of 1 to 10, Is the application easy to use?
3. On a scale of 1 to 10, Is the application useful?
4. On a scale of 1 to 10, Is the application’s content relevant for daily medical practice?
5. Which section in the application is most useful?
   1. Admission
   2. Documents
   3. Videos
   4. Laboratory values
6. Which content would you like to see more of?
   1. More clinical guidance protocols
   2. More clinical scores
   3. More videos of procedural skills
7. Please write if you have any comments and/ suggestions (Free text)
